# Trem2 Enhances Demyelination in the *Csf1r*^+/−^ Mouse Model of Leukoencephalopathy

**DOI:** 10.3390/biomedicines11082094

**Published:** 2023-07-25

**Authors:** Fabrizio Biundo, Violeta Chitu, Şölen Gökhan, Edward Chen, Jude Oppong-Asare, E. Richard Stanley

**Affiliations:** 1 Department of Developmental and Molecular Biology, Albert Einstein College of Medicine, Bronx, NY 10461, USA; 2 Institute for Brain Disorders and Neural Regeneration, Department of Neurology, Albert Einstein College of Medicine, Bronx, NY 10461, USA

**Keywords:** HDLS, ALSP, CSF-1 receptor, TREM2, demyelination, corpus callosum

## Abstract

Colony-stimulating factor-1 receptor (CSF-1R)-related leukoencephalopathy (CRL) is a neurodegenerative disease that triggers early demyelination, leading to an adult-onset dementia. Triggering receptor expressed on myeloid cells-2 (TREM2) is a microglial receptor that promotes the activation of microglia and phagocytic clearance of apoptotic neurons and myelin debris. We investigated the role of Trem2 in the demyelination observed in the *Csf1r*^+/−^ mouse model of CRL. We show that elevation of *Trem2* expression and callosal demyelination occur in 4–5-month-old *Csf1r*^+/−^ mice, prior to the development of symptoms. Absence of *Trem2* in the *Csf1r*^+/−^ mouse attenuated myelin pathology and normalized microglial densities and morphology in the corpus callosum. *Trem2* absence also prevented axonal degeneration and the loss of cortical layer V neurons observed in *Csf1r*^+/−^ mice. Furthermore, the absence of Trem2 prevented the accumulation of myelin-derived lipids in *Csf1r*^+/−^ macrophages and reduced the production of TNF-α after myelin engulfment. These data suggest that TREM2 contributes to microglial dyshomeostasis in CRL.

## 1. Introduction

In the brain, colony-stimulating factor-1 (CSF-1) receptor (CSF-1R) is expressed on microglia [1,2] and is regulated by two neuronally expressed cognate ligands, CSF-1 and interleukin-34 (IL-34) [3] (reviewed in [4]). The development of microglia from yolk sac progenitors and their proliferation and survival in the adult brain is dependent on the CSF-1R [4]. Dominant inactivating mutations in the intracellular kinase domain of the *CSF1R,* or *CSF1R* haploinsufficiency, cause CSF-1-receptor-related leukoencephalopathy (CRL), formerly known as hereditary diffuse leukoencephalopathy with spheroids (HDLS), or adult-onset leukoencephalopathy with axonal spheroids and pigmented glia (ALSP) [5]. CRL is characterized by cognitive and motor impairments, dementia, depression, anxiety and other behavioral deficits [6]. Magnetic resonance imaging of CRL patient brains reveals patchy cerebral white matter lesions, primarily in the frontal and parietal lobe, with thinning of the corpus callosum and enlargement of the lateral ventricles [7,8]. The pathology includes loss of myelin, axonal swelling, degeneration and neuronal cell death [7,9,10,11,12,13,14]. Microgliosis occurs in the early stages of disease when it correlates with the peak of axonal swelling [7,9], while in later stages uneven distribution of microglia with areas of both reduced and increased densities can be found [15]. Furthermore, transcriptomic profiling has provided evidence of loss of microglial homeostatic phenotype [16]. Currently, there are no effective treatment options for CRL. 

We have validated the heterozygous *Csf1r*^+/−^ mouse as a model of CRL [14,17]. This model reproduces the patchy microgliosis, demyelination and neurodegeneration characteristic of the disease and produces behavioral deficits consistent with the motor and cognitive deficits observed in CRL [14]. Transcriptomic profiling of microglia isolated from affected *Csf1r*^+/−^ mice indicates a loss of homeostasis and acquisition of pro-oxidant and demyelinating phenotypes [18]. Importantly, we have shown that microglial *Csf1r* haploinsufficiency is sufficient for disease pathogenesis [19], supporting the concept that CRL is a primary microgliopathy [18].

The microglial phagocytic receptor, TREM2 (triggering receptor expressed on myeloid cells 2) is a lipid receptor that recognizes lipidated ApoE and various phospholipids, including some abundant in myelin (e.g., sulfatides, sphingomyelin and galactosyl ceramide) and phosphatidylserine, which is exposed by damaged neurons and glial cells [20,21,22,23,24]. While TREM2 is dispensable for the phagocytic clearance of apoptotic cells [22], it has a modest contribution to the clearance of myelin debris at low concentrations of CSF-1, but is dispensable at high concentrations [24]. Nevertheless, TREM2 is activated by myelin-derived phospholipids [22,24] and regulates the storage and processing of myelin-derived lipids [24,25]. Furthermore, a TREM2/ApoE pathway contributes to the switch from homeostatic to neurodegenerative microglia phenotype after phagocytosis of apoptotic neurons [26].

Both CSF-1R and TREM2 signal to spleen tyrosine kinase (Syk) via a common adapter protein, DNAX-activating protein of 12 (DAP12) [22,24,27,28], and mutations in either receptor, or in DAP12, cause strikingly similar microgliopathies associated with frontotemporal demyelination and neuronal loss [6,19,29,30,31]. Furthermore, both CSF-1R and TREM2 were coimmunoprecipitated from cells in which they are overexpressed, and in microglial cultures, CSF1R knockdown increased *Trem2* mRNA levels, while CSF1R expression was increased in *Trem2*-deficient microglia [32]. Thus, studies of the contribution of TREM2 to CRL are important to our understanding of how disruption of the crosstalk between TREM2 and the CSF-1R might perturb microglial homeostatic functions. 

*TREM2* expression is elevated in the white matter of CRL patients and in aged *Csf1r*^+/−^ mice [18], but it is not clear whether and how TREM2 might contribute to CRL pathology. Studies in different settings indicate that Trem2 could have protective or deleterious roles in neurodegenerative disease development (reviewed in [33]). Following massive demyelination induced by cuprizone, microglia of *Trem2*^−/−^ mice exhibit defects in lipid metabolism, leading to oxidative stress [24], suggesting a protective role. However, studies of aging indicate that during physiologic, gradual demyelination, TREM2 promotes oxidative stress [34]. Thus, it is possible that increased *Trem2* expression in *Csf1r*^+/−^ mice will exacerbate their pathology and oxidative stress. Our preliminary observation that cerebral *Trem2* expression is elevated in young *Csf1r*^+/−^ mice prompted us to investigate whether Trem2 plays a beneficial or detrimental role in disease-related demyelination in this model. 

## 2. Materials and Methods

### 2.1. Ethics Statement

All in vivo experiments were performed in accordance with the National Institutes of Health regulations on the care and use of experimental animals and approved by the Institutional Animal Care and Use Committees of Albert Einstein College of Medicine.

### 2.2. Mouse Strains, Breeding and Maintenance

*Csf1r*^+/−^ mice [35] backcrossed more than 10 generations to C57BL/6J mice (RRID: IMSR JAX:000664) were crossed with *Trem2*-deficient mice on C57BL/6J background (*Trem2^em2Adiuj^*) [36], obtained from Jackson Laboratories, to create the required genotypes. *Csf1r*^+/−^ mice were genotyped as described previously [35] and *Trem2*-deficient mice were genotyped using the following primers: Common 5′-TCA GGG AGT CAG TCA TTA ACC A–3′; WT Rev 5′AGT GCT TCA AGG CGT CAT AAG T-3′; Mutant Rev 5′-CAA TAA GAC CTG GCA CAA GGA-3′. Cohorts were developed from the progeny of matings of *Csf1r*^+/−^ to *Trem2*^−/−^ mice, randomized with respect to the litter of origin. At 3 months of age, they were transferred from a breeder diet (PicoLab Rodent Diet 20 5058) to a lower-fat maintenance diet (PicoLab Rodent Diet 20 5053). The age and sex of mice used in each experiment are indicated in the figures. 

### 2.3. Gene Expression in Mouse Brains 

RNA was extracted from the anterior motor cortex and corpus callosum of mice as described [17]. cDNA was prepared using a Super Script III First Strand Synthesis kit (Invitrogen, Carlsbad, CA). qPCR was carried out utilizing SYBR Green in an Eppendorf Realplex II thermocycler. Beta actin was used as a housekeeping gene control. The primers for mouse genes used were as follows: *Csf1r* (Fw: 5′-GCAGTACCACCATCCACTTGTA-3′; Rev: 5′-GTGAGACACTGTCCTTCAGTGC-3′); *Trem2* (Fw: 5′-GACCTCTCCACCAGTTTCTCC-3′; Rev: 5′-TACATGACACCCTCAAGGACTG-3′) [37]; *Actb* (Fw: 5′-AGAGGGAAATCGTGCGTGAC-3′; Rev: 5′-CAATAGTGATGACCTGGCCGT-3′).

### 2.4. Ultrastructural Studies

Callosal sections were obtained as described [17] and examined with transmission electron microscopy using a JEOL 1400 transmission electron microscope at 2000× magnification. Ten randomly selected microscopic fields were imaged for each mouse, each field covering an area of 113 μm^2^ (13 μm × 8.7 μm). G-ratios, the ratio of the mean diameter of the axon over the mean diameter of the myelinated fiber, were determined on 200 randomly chosen fibers per mouse (4–8 mice/ genotype) using Image J software (imagej.net). Age-related ultrastructural changes were identified according to the description provided by Peters and Sethares [38] and quantified in 10 different microscopic fields/mouse (8 mice/genotype; average neurons/genotype: *wt*: 1006 ± 55.43, range 851–1261; *Csf1r*^+/−^: 1094 ± 33.49, range 969–1260; *Trem2*^−/−^; *Csf1r*^+/−^: 1095 ± 63.33, range 780–1384; *Trem2*^−/−^: 1028 ± 47.15, range 817–1284).

### 2.5. Immunohistochemistry and Data Analysis

Brain slices were prepared and immune-stained as described previously [17,19]. Staining and quantifications were carried out on one 30 μm thick section per mouse, unless otherwise stated. Brain tissue sections were chosen from matched anatomical regions to ensure consistency and to avoid bias. Sections were incubated overnight at 4 °C with primary antibodies directed to: ionized calcium binding adaptor molecule 1 (Iba1, 1:500) (rabbit IgG; Wako Chemicals RRID: AB_839504), neuronal nuclei (NeuN, 1:500) (mouse IgG, Millipore RRID:AB_2149209) or anti-myelin basic protein (Smi99, 1:500) (mouse IgG BioLegend RRID:AB_2564741). After incubation with primary antibodies, the sections were incubated with secondary antibodies conjugated to either Alexa 488 or Alexa 594 (1:1000) (Life Technologies, Grand Island, NY, USA) at room temperature for 1 h. Fluoromyelin staining (1:350, 30 min) was carried out according to the manufacturer’s (Molecular Probes, Inc., Molecular Probes, Eugene, OR, USA) instructions. A Nikon Eclipse TE300 fluorescence microscope with NISElements D4.10.01 software was used to capture images. Cell numbers were counted manually in anatomically matched regions. The intensity of fluoromyelin staining was evaluated by ImageJ in the supraventricular region of the corpus callosum in an area of ~0.1 mm^2^ (880 μm × 115 μm), using two different sections for each mouse. Adobe Photoshop CS4 was used to crop images and to adjust their brightness, contrast and color balance. Morphometric analysis of microglia was performed on skeletonized images derived from confocal maximum-intensity projections of Iba-1-stained tissue sections using FIJI as described [39]. For this purpose, the images were obtained using a Leica SP5 Confocal microscope. An average of 17.97 ± 3.8 Iba1+ cells were analyzed in each cortical field and 13.5 ± 3.8 Iba1+ cells in each corpus callosum field. Fields were selected and imaged in anatomically comparable regions (i.e., supraventricular corpus callosum and the overlaying motor cortex) by a blinded operator.

### 2.6. Macrophage Preparation and Myelin Challenge

Macrophages were prepared from bone marrow cells cultured in the presence of 140 ng/mL human recombinant CSF-1 (a gift from Chiron Corporation, Emeryville, CA, USA) as previously described [40]. Myelin debris was prepared from brains of wt C57BL6 mice using the protocol of Larocca et al. [41]. For phagocytosis assays, the myelin was fluorescently labeled using the LIVE-DEAD Green amine-reactive dye (Thermo Fisher Scientific, Parsippany, NJ, USA). Unlabeled myelin was used in lipid droplet assays.

For phagocytosis and lipid droplet assays, 5 × 10^5^ macrophages were plated in 60 mm diameter Petri dishes in 3 mL culture medium (αMEM (Corning, Manassas, VA, USA) supplemented with 10% (*v*/*v*) fetal calf serum, 140 ng/mL human recombinant CSF-1, 2 mM L-glutamine, 100 U/mL streptomycin, 100 U/mL penicillin (ATCC, Manassas, VA, USA) and EmbyoMAx nucleosides (Sigma Millipore, St. Louis, MO, USA)). After overnight incubation, the medium was removed, and the cells were stimulated for 3 h (phagocytosis assay) or 24 h (lipid droplet assay) by adding 3 mL of pre-warmed cell culture medium containing 1 mg/mL myelin debris, or were left unstimulated. Following incubation, the cell culture media was removed, and the cells rinsed with ice-cold PBS and harvested by scraping in PBS. The cells were stained with 1:100 Rat anti-mouse CSF-1R-APC (Clone AFS98, BioLegend, San Diego, CA, USA) for 15 min at RT, washed with FACS buffer (1% FCS, 2 mM EDTA, 25 mM HEPES in PBS), then pelleted by centrifugation at 400× *g* for 5 min. Lipid droplets were labeled by resuspending the cells in PBS with 0.5 μg/mL BODIPY 493/503 (Cayman Chemical Company, Ann Arbor, MI, USA) and incubated for 15 min at RT. Dead cells were excluded by staining with 7AAD (BD Pharmingen, San Diego, CA, USA). Cells were then washed two times with FACS buffer and resuspended in FACS buffer with 1 U/mL DNase I (Invitrogen, Carlsbad, CA, USA). Fluorescence was examined with flow cytometry using a FACSAria II (BD Biosciences, Franklin Lakes, NJ, USA) cytometer and the data were analyzed using FloJo (v10).

### 2.7. Cytokine Measurements

Macrophages were harvested by scraping, resuspended at 2.5 × 10^5^ cells/mL in cell culture medium and plated in 96 well tissue culture plates (200 μL/well) in duplicate overnight. The medium was then removed, and the cells were stimulated by adding 200 μL of cell culture medium containing 1 mg/mL myelin debris or left unstimulated. After 24 h incubation, media were removed and tested for TNF-α content by ELISA (eBioscience, San Diego, CA, USA). To control for variations in cell numbers resulting from differences in plating efficiency or proliferation, the cells were rinsed and stained with DAPI, and the cell numbers in each well were determined using a standard curve. The cytokine concentration data were normalized for variations in cell density. 

### 2.8. Statistical Analyses

GraphPad Prism 8 (GraphPad, La Jolla, CA, USA) was used for statistical analyses. Data were checked for outliers (Grubbs’ method) and tested for Gaussian distribution with the Shapiro–Wilk and the Kolmogorov–Smirnov normality tests. Unless otherwise indicated, the screened data were analyzed by analysis of variance (one- or two-way ANOVA) followed by the Benjamini, Krieger and Yekutieli multiple comparison test. Data showing non-Gaussian distributions were analyzed using the Mann–Whitney U test and the Kruskal–Wallis test followed by Dunn’s multiple comparison test. The level of significance was set at *p* < 0.05. Figure legends indicate the sample sizes for each experiment.

## 3. Results

### 3.1. Callosal Demyelination is an Early Feature of Csf1r^+/−^ Mouse Pathology

**Figure 1:**
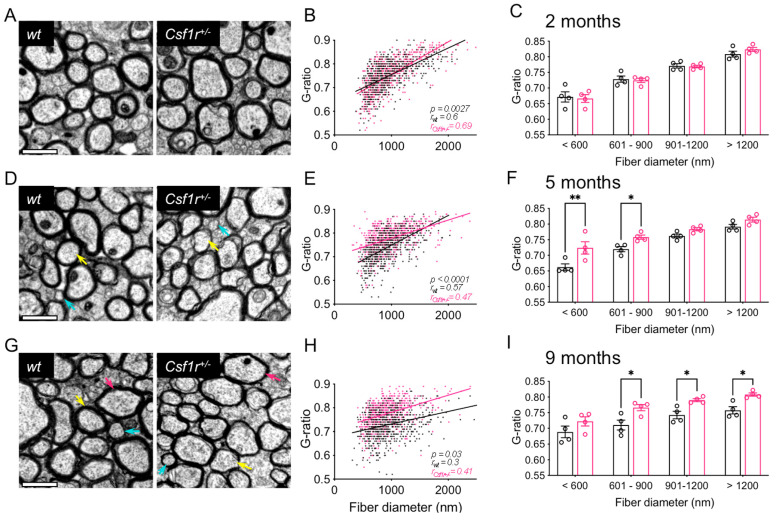
Time course of demyelination in *Csf1r*^+/−^ mice. (**A**,**D**,**G**) Callosal cross-sections from 2-,5- and 9-month-old mice, respectively (2 males and 2 females per each genotype and age). Arrows indicate examples of small (turquoise), medium (yellow) and large (magenta) fibers. Scale bars, 1 μm. (**B**,**E**,**H**) Scatter plot graphs displaying the comparison of G-ratio values for individual fibers between *wt* and *Csf1r*^+/−^ mice at 2, 5 and 9 months of age, respectively (*wt*, black; *Csf1r*^+/−^, magenta). The *p* values on the charts reflect the significance of differences in slopes of the linear regression curves and the r values indicate the goodness-of-fit of the linear regression to the data. (**C**,**F**,**I**) Average G-ratio values for different fiber diameters in *wt* and *Csf1r*^+/−^ mice at 2-, 5- and 9 months of age, respectively (two-way ANOVA, Benjamini, Krieger and Yekutieli). Data are presented as means ± SEM (4 mice/genotype; *, Adjusted *p* value (q) < 0.05; **, q < 0.01).

Previous studies have shown that an increase in the G-ratio of callosal axons indicative of demyelination followed by remyelination is evident as early as 11 months of age in *Csf1r*^+/−^ mice [17]. To determine the time course of this demyelination, we examined cross-sections of corpora callosa obtained from *Csf1r*^+/−^ and control *wt* mice at 2-, 5- and 9 months of age by transmission electron microscopy. Callosal fiber cross-sections were used to calculate axonal G-ratios, the ratio of the inner to the outer diameter of a fiber. An increase in the G-ratio in the axons of *Csf1r*^+/−^ mice compared with *wt* mice was first noticeable by 5 months of age (Figure 1A,D,G). As shown in the scatter (Figure 1B,E,H) and bar (Figure 1C,F,I) plots, significantly increased G-ratios were first evident in the small (<900 nm diameter) fibers in 5-month-old mice (Figure 1E,F), while the higher diameter fibers did not show significant changes until 9 months of age (Figure 1H,I). As behavioral deficits in *Csf1r*^+/−^ mice are first detected at 7 months of age, callosal demyelination is the earliest indicator of disease.

### 3.2. Cerebral Trem2 Expression Is Elevated in Csf1r^+/−^ Brains by 4 Months of Age 

**Figure 2:**
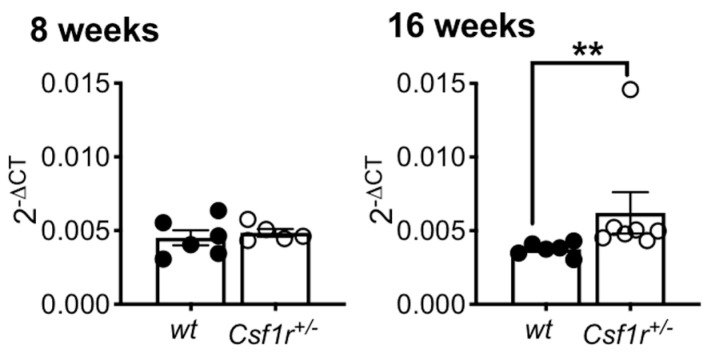
Increased expression of *Trem2* mRNA in the anterior white and grey matter of the motor cortex of *Csf1r*^+/−^ mice by 4 months of age. Means ± SEM, 6–7 mice/genotype, **, *p* < 0.01, Mann–Whitney U test.

We have previously shown that TREM2 expression is elevated in the microglia of 20-month-old *Csf1r*^+/−^ mice and the brains of CRL patients [18]. Given the evidence of demyelination at 5 months of age and the importance of microglial TREM2 in promoting the clearance of apoptotic neurons and myelin debris, we compared the expression of *Trem2* mRNA in young *Csf1r*^+/−^ and control *wt* mice at 2 and 4 months of age (Figure 2). By 2 months of age, in the absence of detectable callosal demyelination, there was no difference in *Trem2* expression between *Csf1r*^+/−^ and *wt* mice. However, by 4 months of age, *Trem2* expression was significantly increased (Figure 2). 

### 3.3. Trem2 Deletion Attenuates Callosal Demyelination in 9-Month-Old Csf1r^+/−^ Mice

**Figure 3:**
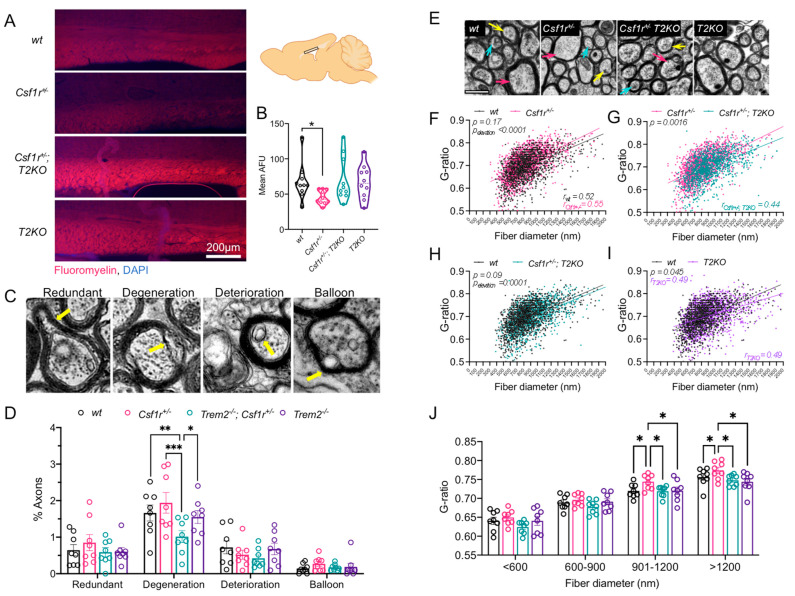
*Trem2* deletion in *Csf1r*^+/−^ mice improves myelination. (**A**) Fluoromyelin staining of the corpus callosum. The schematic on the right side shows the area examined. (**B**) Quantitation of fluoromyelin staining (2 sections per mouse; Kruskal–Wallis test, Dunn’s multiple comparisons). (**C**,**D**) *Trem2* deletion in *Csf1r*^+/−^ mice attenuates myelin degeneration. (**C**) Representative images of age-induced myelin pathologies. Arrows indicate type of pathology. Scale bar, 0.5 μm, applies to all panels. (**D**) Quantification of myelin alterations (two-way ANOVA, Benjamini, Krieger and Yekutieli, means ± SEM, 7–8 mice/genotype; *, q < 0.05; **, q < 0.01; ***, q < 0.001). (**E**–**J**) Absence of *Trem2* prevents the thinning of callosal myelin in *Csf1r*^+/−^ mice. (**E**) Callosal cross-sections from 9-month-old mice. Scale bar, 1 μm, applies to all panels. (**F**–**I**) Scatter plot graphs displaying the comparison of G-ratio values for individual fibers between *wt* and *Csf1r*^+/−^ (**F**), *Csf1r*^+/−^ and *Csf1r*^+/−^; *T2KO* (**G**), *wt* and *Csf1r*^+/−^; *T2KO* (**H**), and between *Csf1r*^+/−^ and *Trem2*^−/−^ mice (**I**). The *p* values on the charts reflect the significance of differences in slopes of the linear regression curves. The p_elevation_ values indicate differences in elevation between curves. The r values indicate the goodness-of-fit of the linear regression to the data. (**J**) Average G-ratio values for different fiber sizes (4 males and 4 females per genotype, one-way ANOVA, Benjamini, Krieger and Yekutieli, Data± SEM, *, q < 0.05; **, q < 0.01; ***, q < 0.001).

To investigate the role of Trem2 in the callosal demyelination in *Csf1r*^+/−^ mice, we developed a cohort comprised of the following genotypes: *Csf1r*^+/+^; *Trem2*^+/+^ (*wt*), *Csf1r*^+/−^; *Trem2*^+/+^(*Csf1r*^+/−^), *Csf1r*^+/−^; *Trem2*^−/−^; (*Csf1r*^+/−^; *T2KO*) and *Trem2*^−/−^; *Csf1r*^+/+^ (*T2KO*). Mice were euthanized at 9 months of age and myelination was examined histologically and ultrastructurally. We observed decreased fluoromyelin staining in the corpus callosum of the *Csf1r*^+/−^ mice that was prevented by *Trem2* deletion (Figure 3A,B). Similar data were obtained with MPB staining (Supplementary Figure S1). Analysis of the myelin pathology indicated that at this age there were no significant increases in the myelin redundancy, degeneration, deterioration or balloon formation in *Csf1r*^+/−^ compared with *wt* mice (Figure 3C,D). However, *Trem2* deletion in *Csf1r*^+/−^ mice significantly reduced the myelin degeneration (Figure 3D). Consistent with this, ultrastructural examination of cross-sections (Figure 3E–I) showed that all *Csf1r*^+/−^ fibers tended to have higher G-ratios than wild-type fibers, with a significant elevation in medium-sized fibers (901–2000 nm diameter, Figure 3J). Loss of *Trem2* had a beneficial effect, decreasing the G-ratio observed in two of the four *Csf1r*^+/−^ fiber size classes (Figure 3F–J). These results suggest that the upregulation of *Trem2* contributes to callosal demyelination in *Csf1r*^+/−^ mice.

### 3.4. Trem2 Deficiency Attenuates Callosal Axonal Degeneration and Loss of Layer V Neurons in 9-Month-Old Csf1r^+/−^ Mice

**Figure 4:**
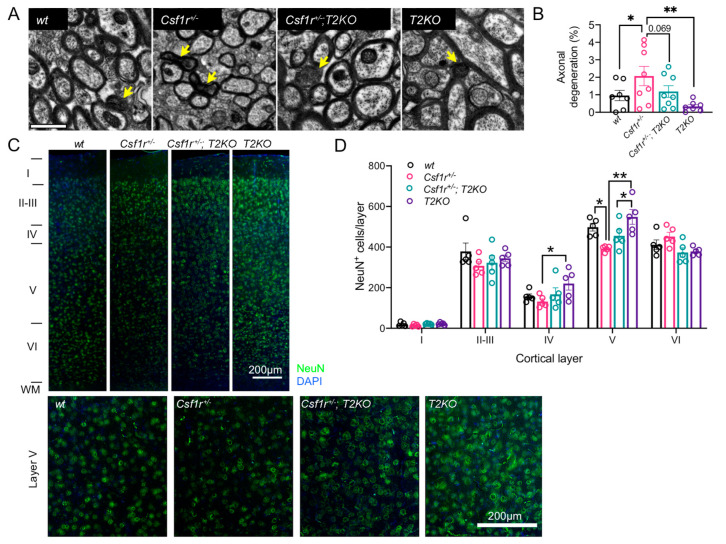
Trem2 deletion in *Csf1r*^+/−^ mice attenuates axonal degeneration and neuronal loss. (**A**,**B**) Attenuation of axonal degeneration by *Trem2* deficiency. (**A**) Callosal cross-sections from 9-month-old mice showing dark axons (arrows). Scale bar, 1 μm, applies to all panels. (**B**) Quantification of axonal degeneration (one-way ANOVA, Benjamini, Krieger and Yekutieli, ± SEM, 8 mice/genotype; *, q < 0.05; **, q < 0.01). (**C**) *Trem2* deficiency attenuates the loss of cortical layer V neuron loss. (**D**) Quantification of cortical neuronal changes. Average NeuN+ cells per layer (two-way ANOVA, Benjamini, Krieger and Yekutielit, ± SEM, *, q < 0.05; **, q < 0.01).

To address the effect of *Trem2* deletion on neurodegeneration, we first examined axonal degeneration in the corpus callosum by scoring dark axons (Figure 4A). Axonal degeneration was increased in *Csf1r*^+/−^ mice and reduced to *wt* levels by deletion of *Trem2* in *Csf1r*^+/−^ mice (Figure 4B). The cell bodies of callosal projection neurons reside primarily in neocortical layers II/III and V. Previous studies have shown that there is selective loss of NeuN^+^ mature neurons in cortical layer V of *Csf1r*^+/−^ mice at 11 months of age and older [18]. Analysis of the distribution of NeuN^+^ neurons among cortical layers also detects the loss of layer V neurons in 9-month-old *Csf1r*^+/−^ mice, a phenotype that is also slightly attenuated by the loss of *Trem2* (Figure 4C,D). These data indicate that the deletion of *Trem2* is neuroprotective in *Csf1r*^+/−^ mice.

### 3.5. Trem2 Ablation Prevents the Increase in Microglial Density and Alterations in Their Morphology in Csf1r^+/−^ Mice 

**Figure 5:**
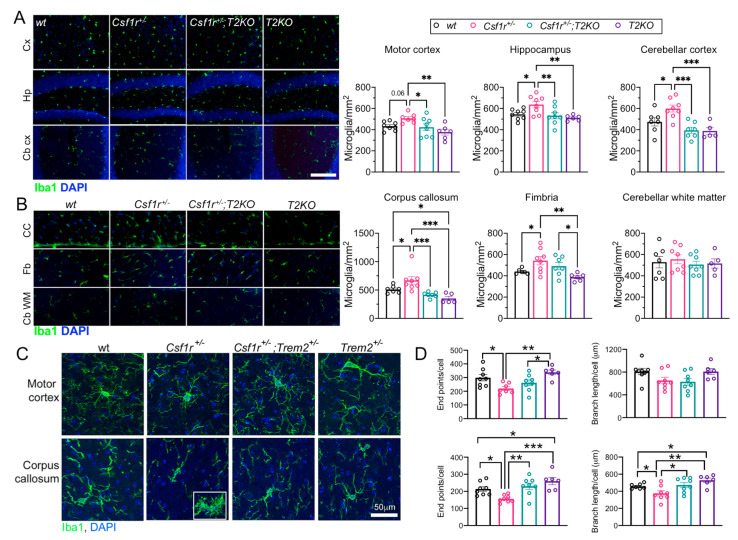
Trem2 deletion normalizes microglia densities and morphology in *Csf1r*^+/−^ mice (**A**,**B**). Illustration of Iba1+ cell densities (green) (left panels) and quantitation (right panels) in white matter and cortical and subcortical brain regions. (**A**) Cortical and subcortical regions. Cx, primary motor cortex; Hp, hippocampus; Cb Cx, cerebellar cortex. (**B**) Quantitation in the white matter. CC, corpus callosum; Fb, fimbria; CbWM, cerebellar white matter. (One-way ANOVA, Benjamini, Krieger and Yekutieli, means ± SEM, 7–8 mice/genotype; *, q < 0.05; **, q < 0.01; ***, q < 0.001). Scale bar, 100 μm applies to all panels. (**C**) Morphology of microglia in the motor cortex and corpus callosum. Insert: Morphology in a periventricular microglial patch. Scale bar applies to all panels. (**D**) Morphometry of microglia in the motor cortex and corpus callosum. (one-way ANOVA, Benjamini, Krieger and Yekutieli, means ± SEM, 7–8 mice/genotype; *, q < 0.05; **, q < 0.01; ***, q < 0.001).

Monoallelic deletion of *Csf1r* in microglia reproduces all the symptoms of *Csf1r*^+/−^ CRL [19] and *Trem2* is selectively expressed in microglia. Quantitation of Iba1-stained microglia in various brain regions revealed elevated microglial densities in the forebrain white matter (corpus callosum and fimbria), but not in the cerebellar white matter. *Trem2* deletion normalized the forebrain white matter densities only (Figure 5B). Increased densities were also observed in the motor cortex, hippocampus and cerebellar cortex of the *Csf1r*^+/−^ mice, and these were also normalized by *Trem2* deletion (Figure 5A). Examination of the morphological changes in the corpus callosum and cerebral cortex revealed a more amoeboid morphology in the *Csf1r*^+/−^ mice (Figure 5C), particularly in the corpus callosum and in the previously reported [18] periventricular microglial patches (Figure 5C, insert). The extent of process branching was reduced in both these regions in *Csf1r*^+/−^ mice and not significantly different from wt in the *Csf1r*^+/−^; *T2KO* mice (Figure 5D, left panels). Branch length was preserved in the cortex and decreased in the corpus callosum of *Csf1r*^+/−^ mice, a phenotype rescued by TREM2 deficiency (Figure 5D, right panels). These results indicate that *Trem2* contributes to the increased microglial densities in several brain regions in *Csf1r*^+/−^ mice and mediates alterations in microglial morphology that are consistent with an altered activation state.

### 3.6. Trem2 Deficiency Does Not Cause the Upregulation of CSF-1R Expression in Macrophages

**Figure 6:**
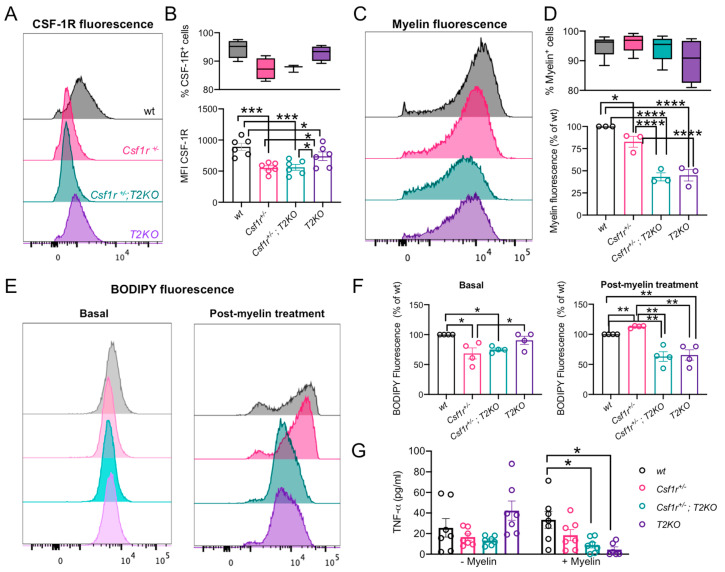
Trem2 deletion reduces myelin engulfment and prevents lipid droplet accumulation in macrophages. (**A**,**B**) TREM2 deficiency does not cause the upregulation of cell surface CSF-1R. (**C**,**D**) *Trem2* deletion reduces myelin engulfment by macrophages. (**E**,**F**) Targeting *Trem2* prevents the excessive accumulation of myelin-derived lipid droplets in *Csf1r*^+/−^ macrophages. (**A**,**C**,**E**) Representative flow cytometry histograms and (**B**,**D**,**F**,**G**) quantification of median fluorescence intensity relative to wt. One-way ANOVA with Benjamini, Krieger and Yekutieli multiple comparison test. (**G**) Effects of TREM2 deficiency on TNF-α production by macrophages in the presence and absence of myelin debris. Two way ANOVA, Benjamini, Krieger and Yekutieli (*, q < 0.05; **, q < 0.01; ***, q < 0.001; ****, q < 0.0001).

Recent work suggested that TREM2 and CSF-1R reciprocally modulate their expression in a negative manner [32]. We therefore reasoned that a mechanism through which *Trem2* deficiency improves CRL could involve the restoration of normal CSF-1R expression in *Csf1r*^+/−^ macrophages. To explore this possibility, we prepared bone-marrow derived macrophages from *Csf1r*^+/−^, *Csf1r*^+/−^; *T2KO* and control mice and evaluated the expression of cell surface CSF-1R at steady state. As shown in Figure 6A,B, *Csf1r*^+/−^ macrophages exhibit a significant decrease in cell surface CSF-1R that is not restored following genetic targeting of *Trem2*. Furthermore, we also failed to detect an increase in cell surface CSF-1R in *Trem2*^−/−^ macrophages.

### 3.7. CSF-1R and TREM2 Have Opposite Effects on the Formation of Myelin-Derived Lipid Droplets in Macrophages 

Since CRL is a demyelinating disease and TREM2 regulates the uptake and processing of myelin debris [24,25], it is important to understand how *Trem2* deficiency affects myelin uptake and processing by *Csf1r*^+/−^ phagocytes. Experiments using bone marrow macrophages show that the majority of macrophages are able to engulf myelin regardless of genotype, but that the amount of myelin engulfed was slightly reduced by loss of a *Csf1r* allele and further decreased by loss of *Trem2* expression (Figure 6C,D). However, despite their reduced capacity to engulf myelin, *Csf1r*^+/−^ macrophages produced more lipid droplets after myelin uptake (Figure 6E,F, right panels). This was not due to an inherent increase in their propensity to store lipids, as under basal conditions, *Csf1r*^+/−^ macrophages contained significantly less lipid droplets than wt counterparts (Figure 6E,F, left panels). Consistent with their reduction in myelin uptake, *Trem2*-deficient macrophages exhibited a substantial reduction in lipid droplet formation following myelin uptake and loss of *Trem2* also decreased the accumulation of lipid droplets in *Csf1r*^+/−^ macrophages (Figure 6E,F). 

Previous studies indicate that myelin can act as an endogenous inflammatory stimulus for macrophages triggering the production of inflammatory cytokines such as TNF-α [42]. Furthermore, the accumulation of lipid droplets in microglia has been reported to create a dysfunctional state and exacerbate the production of inflammatory cytokines [43]. We therefore explored how *Csf1r* and *Trem2* deficiency affect the production of TNF-α. Macrophages were incubated in the presence or absence of myelin for 24h. While the production of TNF-α by *Csf1r*^+/−^ macrophages was comparable to *wt* in both conditions, *Trem2* deficiency suppressed the production of TNF-α in the presence of myelin debris (Figure 6G).

## 4. Discussion

During physiologic, gradual demyelination, in aging mice, TREM2 promotes microgliosis and increases the expression of complement components, markers of oxidative stress and the accumulation of oxidized lipids in microglia [34]. Furthermore, there is evidence that a TREM2/ApoE pathway mediates a switch from a homeostatic to a neurodegenerative microglia phenotype after phagocytosis of apoptotic neurons [26,34]. Analysis of microglial gene expression in *Csf1r*^+/−^ mice with advanced disease predicts maladaptive microglial functions and pathways that trigger oxidative stress and demyelination [18]. *Trem2* expression was also elevated, together with its downstream target *Cst7*, a marker of demyelinating microglia [44,45,46]. Together, these data suggested that TREM2 might contribute to the establishment of a dyshomeostatic state in *Csf1r*^+/−^ microglia. In the present study, we confirm this hypothesis. We show that callosal demyelination is an early pathology in *Csf1r*^+/−^ disease that is associated with an increase in *Trem2* expression. *Trem2* deficiency reduces callosal demyelination and neurodegeneration, that are central to the pathology of this disease. We also show that *Trem2* deletion prevents the increase in microglial density and the changes in microglial morphology. Our results suggest that, in contrast to the protective role of Trem2 observed following recovery from rapid, massive demyelination induced by cuprizone [24], in conditions involving persistent, low-level myelin degeneration and demyelination such as aging [47,48] and CRL (Figure 1), Trem2 has a deleterious effect. These observations prompted us to investigate how *Csf1r* and *Trem2* deficiencies, alone or in combination, affect myelin engulfment and processing by macrophages. We show that while both *Csf1r* heterozygosity and *Trem2* deficiency reduce the engulfment of myelin, they have opposite effects on the processing of myelin-derived lipids. While *Csf1r* heterozygosity enhances, *Trem2* deficiency inhibits the storage of myelin-derived lipids in lipid droplets. Lipid-droplet-laden microglia accumulate in the aging brain and have been reported to exhibit a transcriptional profile consistent with a proinflammatory state [43]. We also find that although the slight increase in lipid droplets in *Csf1r* heterozygous macrophages did not trigger an overt inflammatory state, the decreased accumulation of lipid droplets in *Trem2*-deficient macrophages correlates with decreased production of TNF-α following engulfment of myelin. These data suggest that, by reducing the amount of myelin engulfed, Trem2 deficiency might prevent the acquisition of a dysfunctional state in phagocytes. Indeed, an independent study [49] demonstrates that myelin shedding burdens the clearance function of microglia contributing to the accumulation of lipofuscin within lysosomes and age-associated activation.

Our investigation used bone-marrow-derived macrophages, which are naïve with respect to myelin exposure. Although after the first challenge there was only a mild increase in the propensity of *Csf1r* heterozygous macrophages to store lipids, this finding might be relevant for the pathology of CRL. In middle-aged mice, there is a gradual release of myelin fragments in the extracellular space of the corpus callosum [49]. In addition, the majority of newly generated adult oligodendrocytes (~80%) fail to generate myelin and ultimately die [50]. Thus, unlike other tissue macrophages, brain microglia are constantly involved in the clearance of myelin fragments and oligodendrocytes. Therefore, it is conceivable that, under chronic challenge, even mild deficits in the processing of lipids derived from degenerated myelin or cellular debris could result in a dysfunctional, potentially neuro- or oligo-toxic state. Indeed, Safaiyan et al. [49] have shown that the accumulation of myelin byproducts in microglia is sufficient to induce premature, low-grade activation. In future studies, it will be important to examine in more detail how CSF-1R and TREM2 regulate lipid processing by microglia and the consequences of their individual and combined deficiencies on microglia function.

## 5. Conclusions

These results show that the removal of *Trem2* attenuates pathology in the *Csf1r*^+/−^ mouse model. *Trem2* loss attenuates demyelination, the increase in microglial densities, alteration in microglial morphology, callosal axonal degeneration and the loss of layer V neurons, supporting our conclusion that the loss of *Trem2* is beneficial in this CRL model. While the mechanism involved awaits further investigation, overall, these studies suggest that antagonizing or reducing TREM2 may be therapeutically useful in CRL.

## Data Availability

The data presented in this study are available on request from the corresponding author.

## Supplementary Materials

The following supporting information can be downloaded at: https://www.mdpi.com/article/10.3390/biomedicines11082094/s1.

## References

[1] Akiyama H., Nishimura T., Kondo H., Ikeda K., Hayashi Y., McGeer P.L. (1994). Expression of the receptor for macrophage colony stimulating factor by brain microglia and its upregulation in brains of patients with Alzheimer’s disease and amyotrophic lateral sclerosis. Brain Res..

[2] Raivich G., Haas S., Werner A., Klein M.A., Kloss C., Kreutzberg G.W. (1998). Regulation of MCSF receptors on microglia in the normal and injured mouse central nervous system: A quantitative immunofluorescence study using confocal laser microscopy. J. Comp. Neurol..

[3] Lin H., Lee E., Hestir K., Leo C., Huang M., Bosch E., Halenbeck R., Wu G., Zhou A., Behrens D. (2008). Discovery of a cytokine and its receptor by functional screening of the extracellular proteome. Science.

[4] Chitu V., Gokhan S., Nandi S., Mehler M.F., Stanley E.R. (2016). Emerging Roles for CSF-1 Receptor and its Ligands in the Nervous System. Trends Neurosci..

[5] Rademakers R., Baker M., Nicholson A.M., Rutherford N.J., Finch N., Soto-Ortolaza A., Lash J., Wider C., Wojtas A., DeJesus-Hernandez M. (2011). Mutations in the colony stimulating factor 1 receptor (CSF1R) gene cause hereditary diffuse leukoencephalopathy with spheroids. Nat. Genet..

[6] Konno T., Kasanuki K., Ikeuchi T., Dickson D.W., Wszolek Z.K. (2018). CSF1R-related leukoencephalopathy: A major player in primary microgliopathies. Neurology.

[7] Konno T., Tada M., Tada M., Koyama A., Nozaki H., Harigaya Y., Nishimiya J., Matsunaga A., Yoshikura N., Ishihara K. (2013). Haploinsufficiency of CSF-1R and clinicopathologic characterization in patients with HDLS. Neurology.

[8] Kondo Y., Kinoshita M., Fukushima K., Yoshida K., Ikeda S. (2013). Early involvement of the corpus callosum in a patient with hereditary diffuse leukoencephalopathy with spheroids carrying the de novo K793T mutation of CSF1R. Intern. Med..

[9] Oyanagi K., Kinoshita M., Suzuki-Kouyama E., Inoue T., Nakahara A., Tokiwai M., Arai N., Satoh J.I., Aoki N., Jinnai K. (2017). Adult onset leukoencephalopathy with axonal spheroids and pigmented glia (ALSP) and Nasu-Hakola disease: Lesion staging and dynamic changes of axons and microglial subsets. Brain Pathol..

[10] Coleman M. (2005). Axon degeneration mechanisms: Commonality amid diversity. Nat. Rev. Neurosci..

[11] Beirowski B., Nogradi A., Babetto E., Garcia-Alias G., Coleman M.P. (2010). Mechanisms of axonal spheroid formation in central nervous system Wallerian degeneration. J. Neuropathol. Exp. Neurol..

[12] Alturkustani M., Keith J., Hazrati L.N., Rademakers R., Ang L.C. (2015). Pathologic staging of white matter lesions in adult-onset leukoencephalopathy/leukodystrophy with axonal spheroids. J. Neuropathol. Exp. Neurol..

[13] Zhan F.X., Zhu Z.Y., Liu Q., Zhou H.Y., Luan X.H., Huang X.J., Liu X.L., Tian W.T., Wang S.G., Song X.X. (2020). Altered structural and functional connectivity in CSF1R-related leukoencephalopathy. Brain Imaging Behav..

[14] Chitu V., Gokhan S., Stanley E.R. (2022). Modeling CSF-1 receptor deficiency diseases—How close are we?. FEBS J..

[15] Tada M., Konno T., Tada M., Tezuka T., Miura T., Mezaki N., Okazaki K.I., Arakawa M., Itoh K., Yamamoto T. (2016). Characteristic microglial features in patients with hereditary diffuse leukoencephalopathy with spheroids. Ann. Neurol..

[16] Kempthorne L., Yoon H., Madore C., Smith S., Wszolek Z.K., Rademakers R., Kim J., Butovsky O., Dickson D.W. (2020). Loss of homeostatic microglial phenotype in CSF1R-related Leukoencephalopathy. Acta Neuropathol. Commun..

[17] Chitu V., Gokhan S., Gulinello M., Branch C.A., Patil M., Basu R., Stoddart C., Mehler M.F., Stanley E.R. (2015). Phenotypic characterization of a Csf1r haploinsufficient mouse model of adult-onset leukodystrophy with axonal spheroids and pigmented glia (ALSP). Neurobiol. Dis..

[18] Chitu V., Biundo F., Shlager G.G.L., Park E.S., Wang P., Gulinello M.E., Gokhan S., Ketchum H.C., Saha K., DeTure M.A. (2020). Microglial Homeostasis Requires Balanced CSF-1/CSF-2 Receptor Signaling. Cell Rep..

[19] Biundo F., Chitu V., Shlager G.G.L., Park E.S., Gulinello M.E., Saha K., Ketchum H.C., Fernandes C., Gokhan S., Mehler M.F. (2021). Microglial reduction of colony stimulating factor-1 receptor expression is sufficient to confer adult onset leukodystrophy. Glia.

[20] Kober D.L., Alexander-Brett J.M., Karch C.M., Cruchaga C., Colonna M., Holtzman M.J., Brett T.J. (2016). Neurodegenerative disease mutations in TREM2 reveal a functional surface and distinct loss-of-function mechanisms. Elife.

[21] Sudom A., Talreja S., Danao J., Bragg E., Kegel R., Min X., Richardson J., Zhang Z., Sharkov N., Marcora E. (2018). Molecular basis for the loss-of-function effects of the Alzheimer’s disease-associated R47H variant of the immune receptor TREM2. J. Biol. Chem..

[22] Wang Y., Cella M., Mallinson K., Ulrich J.D., Young K.L., Robinette M.L., Gilfillan S., Krishnan G.M., Sudhakar S., Zinselmeyer B.H. (2015). TREM2 lipid sensing sustains the microglial response in an Alzheimer’s disease model. Cell.

[23] Du H., Grabowski G.A. (2004). Lysosomal acid lipase and atherosclerosis. Curr. Opin. Lipidol..

[24] Nugent A.A., Lin K., van Lengerich B., Lianoglou S., Przybyla L., Davis S.S., Llapashtica C., Wang J., Kim D.J., Xia D. (2020). TREM2 Regulates Microglial Cholesterol Metabolism upon Chronic Phagocytic Challenge. Neuron.

[25] Gouna G., Klose C., Bosch-Queralt M., Liu L., Gokce O., Schifferer M., Cantuti-Castelvetri L., Simons M. (2021). TREM2-dependent lipid droplet biogenesis in phagocytes is required for remyelination. J. Exp. Med..

[26] Krasemann S., Madore C., Cialic R., Baufeld C., Calcagno N., El Fatimy R., Beckers L., O’Loughlin E., Xu Y., Fanek Z. (2017). The TREM2-APOE Pathway Drives the Transcriptional Phenotype of Dysfunctional Microglia in Neurodegenerative Diseases. Immunity.

[27] Otero K., Turnbull I.R., Poliani P.L., Vermi W., Cerutti E., Aoshi T., Tassi I., Takai T., Stanley S.L., Miller M. (2009). Macrophage colony-stimulating factor induces the proliferation and survival of macrophages via a pathway involving DAP12 and beta-catenin. Nat. Immunol..

[28] Cheng Q., Danao J., Talreja S., Wen P., Yin J., Sun N., Li C.M., Chui D., Tran D., Koirala S. (2018). TREM2-activating antibodies abrogate the negative pleiotropic effects of the Alzheimer’s disease variant Trem2(R47H) on murine myeloid cell function. J. Biol. Chem..

[29] Bianchin M.M., Martin K.C., de Souza A.C., de Oliveira M.A., Rieder C.R. (2010). Nasu-Hakola disease and primary microglial dysfunction. Nat. Rev. Neurol..

[30] Paloneva J., Kestila M., Wu J., Salminen A., Bohling T., Ruotsalainen V., Hakola P., Bakker A.B., Phillips J.H., Pekkarinen P. (2000). Loss-of-function mutations in TYROBP (DAP12) result in a presenile dementia with bone cysts. Nat. Genet..

[31] Paloneva J., Manninen T., Christman G., Hovanes K., Mandelin J., Adolfsson R., Bianchin M., Bird T., Miranda R., Salmaggi A. (2002). Mutations in two genes encoding different subunits of a receptor signaling complex result in an identical disease phenotype. Am. J. Hum. Genet..

[32] Cheng B., Li X., Dai K., Duan S., Rong Z., Chen Y., Lu L., Liu Z., Huang X., Xu H. (2021). Triggering Receptor Expressed on Myeloid Cells-2 (TREM2) Interacts with Colony-Stimulating Factor 1 Receptor (CSF1R) but Is Not Necessary for CSF1/CSF1R-Mediated Microglial Survival. Front. Immunol..

[33] Konishi H., Kiyama H. (2018). Microglial TREM2/DAP12 Signaling: A Double-Edged Sword in Neural Diseases. Front. Cell. Neurosci..

[34] Linnartz-Gerlach B., Bodea L.G., Klaus C., Ginolhac A., Halder R., Sinkkonen L., Walter J., Colonna M., Neumann H. (2019). TREM2 triggers microglial density and age-related neuronal loss. Glia.

[35] Dai X.M., Ryan G.R., Hapel A.J., Dominguez M.G., Russell R.G., Kapp S., Sylvestre V., Stanley E.R. (2002). Targeted disruption of the mouse colony-stimulating factor 1 receptor gene results in osteopetrosis, mononuclear phagocyte deficiency, increased primitive progenitor cell frequencies, and reproductive defects. Blood.

[36] Kang S.S., Kurti A., Baker K.E., Liu C.C., Colonna M., Ulrich J.D., Holtzman D.M., Bu G., Fryer J.D. (2018). Behavioral and transcriptomic analysis of Trem2-null mice: Not all knockout mice are created equal. Hum. Mol. Genet..

[37] Jiang T., Yu J.T., Zhu X.C., Tan M.S., Gu L.Z., Zhang Y.D., Tan L. (2014). Triggering receptor expressed on myeloid cells 2 knockdown exacerbates aging-related neuroinflammation and cognitive deficiency in senescence-accelerated mouse prone 8 mice. Neurobiol. Aging.

[38] Peters A., Folger Sethares C. The Fine Structure of the Aging Brain. http://www.bu.edu/agingbrain.

[39] Young K., Morrison H. (2018). Quantifying Microglia Morphology from Photomicrographs of Immunohistochemistry Prepared Tissue Using ImageJ. J. Vis. Exp..

[40] Rolfe A.J., Bosco D.B., Broussard E.N., Ren Y. (2017). In Vitro Phagocytosis of Myelin Debris by Bone Marrow-Derived Macrophages. J. Vis. Exp..

[41] Larocca J.N., Norton W.T. (2007). Isolation of myelin. Curr. Protoc. Cell Biol..

[42] Sun X., Wang X., Chen T., Li T., Cao K., Lu A., Chen Y., Sun D., Luo J., Fan J. (2010). Myelin activates FAK/Akt/NF-kappaB pathways and provokes CR3-dependent inflammatory response in murine system. PLoS ONE.

[43] Marschallinger J., Iram T., Zardeneta M., Lee S.E., Lehallier B., Haney M.S., Pluvinage J.V., Mathur V., Hahn O., Morgens D.W. (2020). Lipid-droplet-accumulating microglia represent a dysfunctional and proinflammatory state in the aging brain. Nat. Neurosci..

[44] Ma J., Tanaka K.F., Shimizu T., Bernard C.C., Kakita A., Takahashi H., Pfeiffer S.E., Ikenaka K. (2011). Microglial cystatin F expression is a sensitive indicator for ongoing demyelination with concurrent remyelination. J. Neurosci. Res..

[45] Keren-Shaul H., Spinrad A., Weiner A., Matcovitch-Natan O., Dvir-Szternfeld R., Ulland T.K., David E., Baruch K., Lara-Astaiso D., Toth B. (2017). A Unique Microglia Type Associated with Restricting Development of Alzheimer’s Disease. Cell.

[46] Colonna M., Brioschi S. (2020). Neuroinflammation and neurodegeneration in human brain at single-cell resolution. Nat. Rev. Immunol..

[47] Peters A. (2002). Structural changes that occur during normal aging of primate cerebral hemispheres. Neurosci. Biobehav. Rev..

[48] Rivera A.D., Pieropan F., Chacon-De-La-Rocha I., Lecca D., Abbracchio M.P., Azim K., Butt A.M. (2021). Functional genomic analyses highlight a shift in Gpr17-regulated cellular processes in oligodendrocyte progenitor cells and underlying myelin dysregulation in the aged mouse cerebrum. Aging Cell.

[49] Safaiyan S., Kannaiyan N., Snaidero N., Brioschi S., Biber K., Yona S., Edinger A.L., Jung S., Rossner M.J., Simons M. (2016). Age-related myelin degradation burdens the clearance function of microglia during aging. Nat. Neurosci..

[50] Hill R.A., Li A.M., Grutzendler J. (2018). Lifelong cortical myelin plasticity and age-related degeneration in the live mammalian brain. Nat. Neurosci..

