# Supplementary figures and images for: Trem2 Enhances Demyelination in the Csf1r+/− Mouse Model of Leukoencephalopathy
Source: Biomedicines. 2023 Jul 25;11(8):2094. doi: 10.3390/biomedicines11082094 (PMC10452898; doi:10.3390/biomedicines11082094)

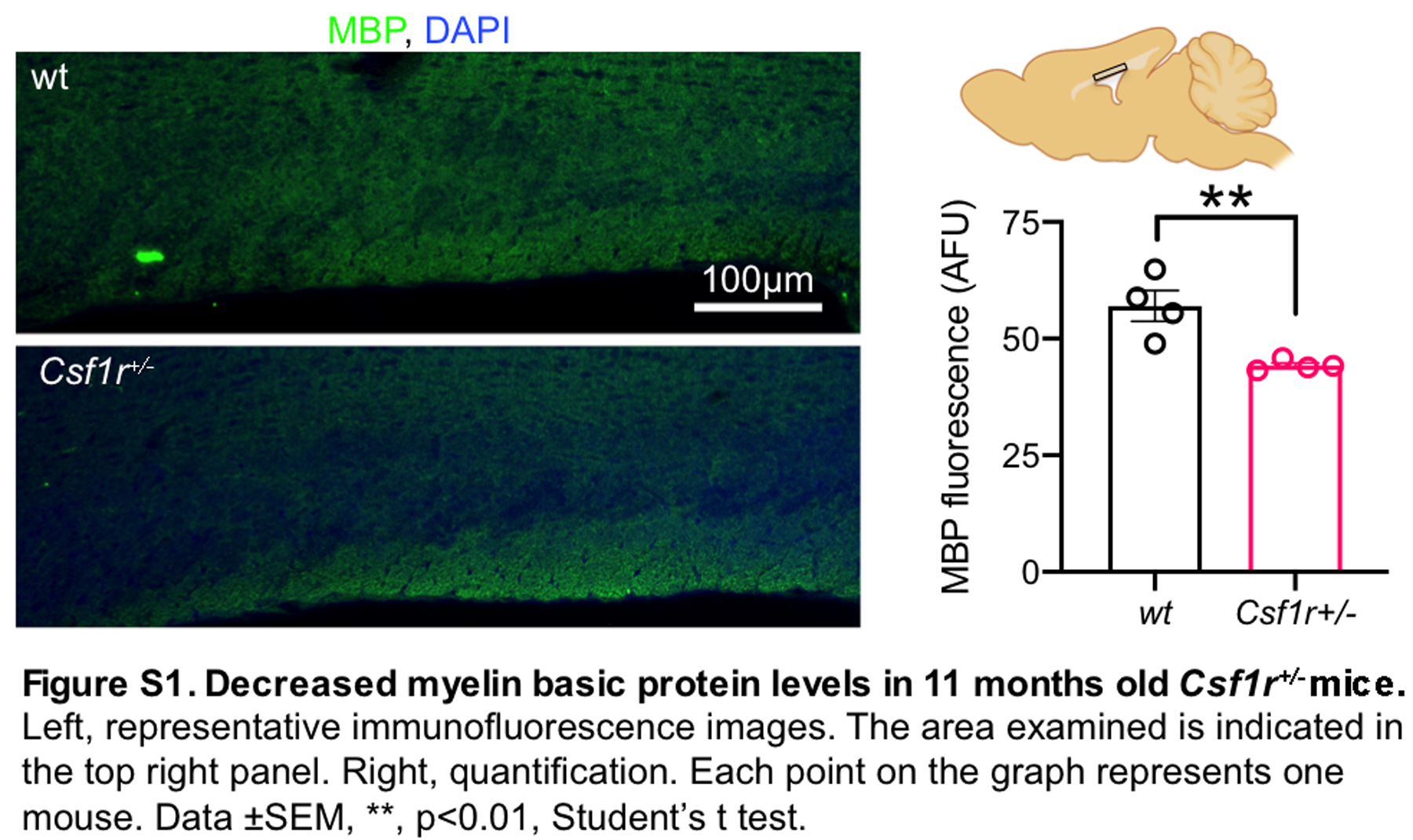

Supplement: Supplementary file 1 [file biomedicines-11-02094-s001.zip › biomedicines-2325443-supplementary.tif]
